# Notes and News

**Published:** 1897-07-17

**Authors:** 


### Notes and News.

(Collected and Communicated.)

Madame Clarens has bequeathed 15,000 francs to the French Academy of Medicine to found an annual prize in medicine.

On Tuesday, July 20th, the medical staff and lecturers of the Dental Hospital of London will hold a conversazione at the Galleries of the Royal Society of British Artists at half-past eight p.m.

Through the will of Mr. Charles Clement Walker, of Lilleshall, charity will receive about £100,000. Local charities, including the Shrewsbury Infirmary, receive immediate bequests.

The foundation-stone of a new wing at the West Kent Hospital has been laid. The building will form a memorial of the sixtieth year of the Queen's reign. The cost is estimated at £2,300.

Princess Louise will open the Passmore Edwards' Convalescent Home for the Metropolitan Hospital at Cranbrook, Kent, on Tuesday, July 20th next, at half-past four p.m.

By the will of Mrs. Gee, widow of the late Robert Gee, lecturer on the diseases of children in the Liverpool Medical Schools, over £7,000 has been bequeathed to the University College, Liverpool, for the purpose of advancing the medical department and promoting the study and research of medical science. It has been decided to institute a Robert Gee Fellowship in Anatomy, value £100, for one year, and four entrance scholarships of £25 for one year each.

The largest annual subscriber to the Prince of Wales's Hospital Fund so far is the Hon. W. W. Astor, who has just paid in to the account at the Bank of England a year's subscription of £1,000. Arrangements have been made with all district messenger offices to supply the Commemoration Hospital Stamps, which serve as receipts for small donations to the hospitals through the Prince of Wales's Hospital Fund.

The annual meeting of the Association for the Oral Instruction of the Deaf and Dumb will be held at 11, Fitzroy Square, on Tuesday, July 20th, at half-past three. Prizes will be distributed, and a demonstration of the system given.

Professor Mikulicz, of Breslau, has adopted fine thread gloves made aseptic whilst performing surgical operations. It is stated that he finds they in no way impede dexterity; in fact, they afford extra facility in grasping tissues.

The new rules under the Cantonments Bill have been published. They apply equally to all contagious diseases, and require that doctors and house-owners shall give information respecting any outbreak of such diseases. Objections to the rules will be considered up to August 10th.

Newcastle is naturally and justly proud that it has raised the whole of the £100,000 necessary to erect the intended new infirmary. Belfast, too, is jubilant over a like achievement on behalf of their proposed Royal Victoria Commemoration Hospital. The magnificent collection of £100,000 has been raised in the short space of six months, an excellent example of generosity, energy, and enthusiasm displayed by Belfast.

At the Medical, Surgical, and Hygienic Exhibition, to be held at the Queen's Hall, Langham Place, from July 26th to the 30th, the Scientific Press will have an exhibit. Amongst the other interesting matters to be seen there will be surgical instruments and appliances, food products, &c., as they were in 1837, and as they are now, the object of this exhibition being to show the progress that has been made during the last 60 years in the manufacture of these and other goods having to do with the science and art of medicine and surgery.

Although we have heard some complaints that definite information as to arrangements is hard to obtain, the International Medical Congress at Moscow is certainly advancing towards preparedness. The railway arrangements leave the intending visitor a fair amount of liberty of action, and provide for a possible holiday when the business of the meeting is over, and a good many interesting excursions and entertainments are on the programme. The usual clinical demonstrations will be given in the clinics, and a small exhibition of surgical instruments will be organised.

The new fever hospital of the Metropolitan Asylums Board at Hither Green was opened by the Prince of Wales on Monday last. The Park Hospital is to contain 548 patients, in 60 wards. The arrangements and general plan is somewhat the same as that of the Brook Hospital at Shooter's Hill. The site is good but a little crowded. The occasion of the opening was taken to make a loyal demonstration at Lewisham, and along the whole route along which the Royal *cortège* passed, and the decorations were both tasteful and lavish. A very large number of guests were invited to attend the ceremony, and the Royal and other visitors expressed themselves much interested with the arrangements of the great hospital.

